# Intravitreal AAV-Delivery of Genetically Encoded Sensors Enabling Simultaneous Two-Photon Imaging and Electrophysiology of Optic Nerve Axons

**DOI:** 10.3389/fncel.2018.00377

**Published:** 2018-10-23

**Authors:** Zoe J. Looser, Matthew J. P. Barrett, Johannes Hirrlinger, Bruno Weber, Aiman S. Saab

**Affiliations:** ^1^Institute of Pharmacology & Toxicology, University of Zurich, Zurich, Switzerland; ^2^Neuroscience Center Zurich, University and ETH Zurich, Zurich, Switzerland; ^3^Carl-Ludwig-Institute for Physiology, University of Leipzig, Leipzig, Germany; ^4^Department of Neurogenetics, Max-Planck-Institute of Experimental Medicine, Göttingen, Germany

**Keywords:** myelinated axons, intravitreal AAV injection, optic nerve recording, two-photon imaging, genetically encoded sensors, ATP-sensor ATeam1.03YEMK

## Abstract

Myelination of axons by oligodendrocytes is a key feature of the remarkably fast operating CNS. Oligodendrocytes not only tune axonal conduction speed but are also suggested to maintain long-term axonal integrity by providing metabolic support to the axons they ensheath. However, how myelinating oligodendrocytes impact axonal energy homeostasis remains poorly understood and difficult to investigate. Here, we provide a method of how to study electrically active myelinated axons expressing genetically encoded sensors by combining electrophysiology and two-photon imaging of acutely isolated optic nerves. We show that intravitreal adeno-associated viral (AAV) vector delivery is an efficient tool to achieve functional sensor expression in optic nerve axons, which is demonstrated by measuring axonal ATP dynamics following AAV-mediated sensor expression. This novel approach allows for fast expression of any optical sensor of interest to be studied in optic nerve axons without the need to go through the laborious process of producing new transgenic mouse lines. Viral-mediated biosensor expression in myelinated axons and the subsequent combination of nerve recordings and sensor imaging outlines a powerful method to investigate oligodendroglial support functions and to further interrogate cellular mechanisms governing axonal energy homeostasis under physiological and pathological conditions.

## Introduction

Information processing in the brain critically relies on electrical signal transmissions along axons interconnecting a long-range network of myriads of neurons. By insulating axons with myelin, oligodendrocytes are essential for rapid saltatory impulse propagation (Huxley and Stämpfli, 1949) and thereby influence the computational speed and efficiency of higher vertebrate nervous systems (Laughlin and Sejnowski, 2003). About 50% of the human brain is composed of white matter tracts, which mainly contain myelinated axons and their associated glial cells. Loss of axonal integrity is detrimental for normal brain function and deficits in white matter energy metabolism, including perturbations in glial metabolic support to myelinated axons (Lee et al., 2012; Saab et al., 2016), are increasingly discussed in the pathogenesis of various neurodegenerative diseases (Nave, 2010a; Iadecola, 2013; Philips and Rothstein, 2017; Saab and Nave, 2017).

To better understand how white matter pathology and axonal degeneration may associate with perturbations in white matter energy homeostasis, more insights into cellular mechanisms regulating axonal metabolite supply and energy metabolism are required. For example, how myelinating oligodendrocytes regulate axonal energy metabolism and maintain axonal integrity under physiological or pathological conditions is still elusive and difficult to investigate. Genetically-encoded optical sensors enable real-time monitoring of cellular metabolite dynamics and are becoming invaluable neurobiological tools to study intercellular mechanisms governing brain energy homeostasis (Hou et al., 2011; San Martin et al., 2014b; Sotelo-Hitschfeld et al., 2015; Mächler et al., 2016; Díaz-Garcia et al., 2017; Trevisiol et al., 2017). The fully myelinated optic nerve is an ideal white matter tract to examine mechanisms of axonal energy metabolism *ex vivo* (Stys et al., 1991; Brown et al., 2001; Tekkök et al., 2005; Saab et al., 2016). A recent study introduced a novel approach to measure activity-dependent axonal ATP homeostasis in acute optic nerve preparations by combining nerve recordings with confocal imaging (Trevisiol et al., 2017). There, axonal sensor expression of the FRET-based ATP sensor ATeam1.03^YEMK^ (Imamura et al., 2009) in optic nerves was achieved by generating a transgenic mouse with a pan-neuronal expression of the ATP sensor (Trevisiol et al., 2017). However, generation of new transgenic mice is time consuming and costly if one would like to expand the repertoire of biosensor measurements in myelinated axons using the acute optic nerve imaging paradigm. Especially with new and improved optical sensors being continuously produced, we wondered whether functional sensor expression in optic nerve axons could be achieved by viral-mediated delivery.

The aim of this study is to demonstrate that intravitreal injection of adeno-associated viral (AAV) vectors carrying genetically encoded sensors is suitable to drive robust and functional sensor expression in optic nerve axons. This allows the analysis of activity-dependent axonal metabolite dynamics in acute optic nerve preparations by combining electrophysiology with two-photon imaging. As a proof of principle, we tested intravitreal injections of AAV vectors with three different serotypes carrying the ATP sensor ATeam1.03^YEMK^ (Imamura et al., 2009) and evaluated axonal sensor expression and activity in our acute optic nerve recording/imaging paradigm. Intravitreal AAV-mediated sensor expression and subsequent two-photon imaging of myelinated axons represents a powerful approach to gain more insights into cellular and molecular mechanisms governing white matter energy metabolism.

## Materials and Methods

### Animals

All animal experiments were approved by the local veterinary authorities in Zurich and were according to the guidelines of Swiss Animal Protection Law, Veterinary Office, Canton Zurich (Animal Welfare Act of 16 December 2005 and Animal Welfare Ordinance of 23 April 2008). Mice had free access to food and water and were kept with an inverted 12 h light/dark cycle. Intravitreal AAV vector injections were performed in 8–10 weeks old wild type mice (C57BL/6J; Charles River) and optic nerve imaging experiments were performed starting from 3 weeks to 4 weeks after the intravitreal injection. Transgenic mice B6-Tg(Thy1.2-ATeam1.03^YEMK^)AJhi (Trevisiol et al., 2017) at the age of 12–15 weeks were also used for imaging experiments. Safety measures for handling AAV viral vectors were according to the institutional biosafety procedures of the University of Zurich.

### Experimental Design

**Figure 1:**
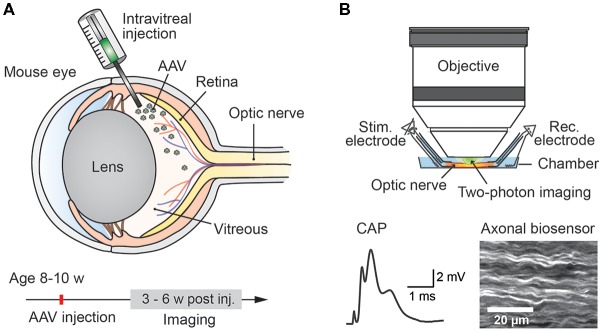
Experimental paradigm: intravitreal adeno-associated viral (AAV) vector injection and subsequent two-photon imaging of genetically encoded biosensors in optic nerve axons. **(A)** Injection of AAV vector carrying genetically encoded sensor into the vitreous humor of the mouse eye at the age of 8–10 weeks. Robust sensor expression in optic nerve axons for imaging experiments is achieved starting from 3 weeks to 4 weeks after the injection. **(B)** Acute optic nerve preparation for combined electrophysiology and two-photon imaging of myelinated axons. Simultaneous monitoring of stimulus-evoked compound action potentials (CAPs) and biosensor activity enables studying activity-dependent axonal metabolite dynamics.

Studying energy metabolism in white matter tracts is challenging, but essential to understand how axonal compartments, which are shielded by myelin sheaths, take up metabolites, regulate their energy resources and maintain functional integrity (Nave, 2010b; Saab et al., 2013; Trevisiol et al., 2017). We describe a novel approach which allows any genetically encoded optical sensor of interest to be rapidly expressed and studied in optic nerve axons of adult mice (Figure 1). Since all optic nerve axons descend from retinal ganglion cells (RGCs) located in the retina, intravitreal injection of AAV vectors enables sensor expression in RGCs and their myelinated axons. Around 3–4 weeks after intravitreal AAV injections, robust sensor expression in optic nerve axons was achieved and nerves were prepared for *ex vivo* recordings and axonal imaging. Electrical nerve stimulations and recordings of compound action potentials (CAPs) were combined with two-photon imaging. We demonstrate the usefulness of this protocol by monitoring axonal ATP level changes following intravitreal AAV-mediated delivery and expression of a FRET-based ATP sensor ATeam1.03^YEMK^ (Imamura et al., 2009). For comparison, we also monitored axonal ATP dynamics in optic nerves from transgenic mice constitutively expressing the same ATP sensor in neuronal/axonal compartments (Trevisiol et al., 2017).

### Intravitreal AAV Injections

**Figure 2:**
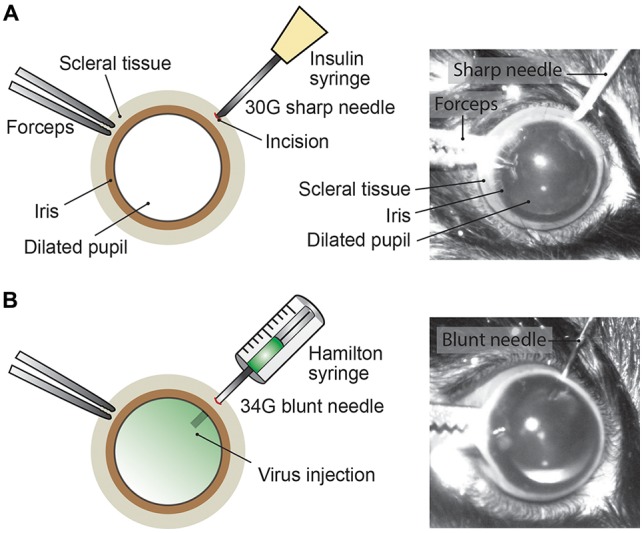
Intravitreal AAV injection procedure. **(A)** Depiction of the mouse eye (frontal view) with a dilated pupil held by the scleral connective tissue with forceps. Using a sharp 30G needle (e.g., insulin syringe) an incision is made into the sclera. **(B)** A Hamilton syringe with a 34G blunt needle is carefully inserted into the incision and the AAV viral suspension is slowly injected into the vitreous. By adding fluorescein dye (green) to the viral suspension, the intravitreal injection can be observed by the homogenous diffusion of the green dye inside the vitreous.

Mice were anesthetized with an intraperitoneal injection of fentanyl (0.05 mg/kg; Fentanyl Sintetica, Sintetica), midazolam (5 mg/kg; Dormicum, Roche), and medetomidine (0.5 mg/kg; Domitor, Orion Pharma) mixed in NaCl (0.9%; Fleischmann et al., 2016). For pupil dilation, first cyclopentolate (1%; Cyclogyl, Alcon) and then phenylephrine (5%; Neosynephrin-POS, URSAPHARM) were each applied topically to the eyes for 1–2 min. Prolonged light exposure to the mouse was avoided whenever possible once pupils were dilated. Eyes were kept moisturized with the transparent Viscotears liquid gel (Alcon) and mouse body temperature was kept stable at 37°C throughout the intravitreal AAV injection procedure (Figure 2). Under microscopic control (SteREO Discovery.V20, Zeiss) an incision was made into the sclera (1–2 mm posterior of the superior limbus) using a sterile, sharp 30-gauge (G) needle (insulin syringe, Omnican 50, Braun; Figure 2A). Great care was taken not to damage the lens or retina. After removing the 30G needle, a 34G blunt needle (12.70mm/pst3, BGB, HA-207434) attached to a microliter Hamilton syringe (10 μl Syringe Model 701) was carefully inserted into the same incision (Figure 2B) and 1–1.5 μl of viral suspension was slowly injected (0.05–0.1 μl/s) into the vitreous. For a better monitoring of the AAV injection into the vitreous, we mixed 1 μl of fluorescein (0.1 mg/ml in PBS) in 10 μl of undiluted viral suspension (see below). Successful injections were observed by the homogenous diffusion of the green fluorescein dye inside the vitreous (Figure 2B). The needle was kept inside the vitreous for 1–2 min after the injection to prevent reflux of viral suspension, before carefully removing the needle. Both eyes were injected. After intravitreal AAV injections, antibiotic eye drops (Ofloxacin 0.3%, Floxal UD, Bausch + Lomb) were applied and mice were injected subcutaneously with the analgesic buprenorphine (0.1 mg/kg, Temgesic, Indivior Schweiz AG). By antagonizing the anesthesia with intraperitoneal administration of the antagonists (a mixture of atipamezole (2.5 mg/kg; Revertor, Virbac) and flumazenil (0.5 mg/kg; Anexate, Roche)) mice typically regained consciousness within 5 min when placed back in the home cage for recovery.

### AAV Viral Vectors

The production, purification and quantification of single-stranded (ss) AAV vectors were performed by the viral vector facility (VVF) of the Neuroscience Center Zurich (ZNZ) as described (Zolotukhin et al., 1999; Paterna et al., 2004). The AAV viral vectors used in this study express the ATP sensor ATeam1.03^YEMK^ (Imamura et al., 2009) under the control of the human synapsin promoter (hSyn1-ATeam1.03^YEMK^-WPRE-hGHp(A)) and three different AAV vector serotypes, ssAAV-2/2, -DJ/2 and -9/2 (Samulski et al., 1989; Gao et al., 2004; Grimm et al., 2008), were generated and tested. Intravitreal AAV injections were performed with undiluted viral suspensions and the physical titers of ssAAV-2/2, -DJ/2 and -9/2 were 6.2 × 10^12^ vg/ml (vector genomes/milliliter), 5.4 × 10^12^ vg/ml and 8.1 × 10^12^ vg/ml, respectively. The VVF repository identifier of the hSyn1-ATeam1.03^YEMK^ AAV vectors is v244 and all AAV serotypes as well as detailed information on AAV production, including vector maps, can be obtained from the VVF homepage^1^.

### Optic Nerve Preparation Procedure

**Figure 3:**
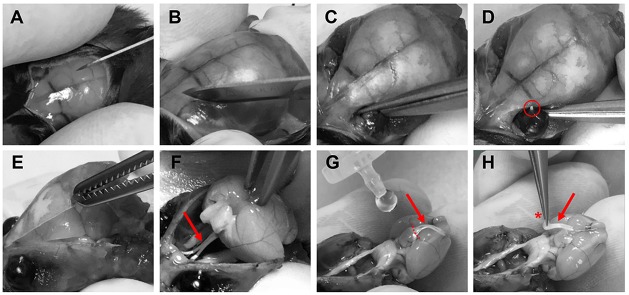
Procedure of optic nerve removal. **(A)** Following anesthesia and decapitation the skin covering the skull is cut longitudinally. **(B,C)** The optic nerves are separated from the eyeballs after removing connective tissue by carefully cutting into the eye socket behind the eyeballs. **(D)** After the separation the exit tips of the nerve (red circle) are visible as white dots at the back of the eyeballs. **(E)** The skull is removed to gain access to the brain. **(F)** The brain is pulled backwards to extract the optic nerves (red arrows **F–H**) from the optical canals. Nerves should come out easily still attached to the chiasm. **(G)** With drops of artificial cerebrospinal fluid (ACSF), nerves are flushed to straighten them, and the chiasm is cut (dashed lines) leaving half of the chiasm still connected to the nerve pair. **(H)** Optic nerves are carefully lifted by holding at the chiasm (red asterisk) and then transferred to the recording chamber.

Optic nerves were prepared for imaging experiments 3–6 weeks after intravitreal AAV vector injections. Mice were deeply anesthetized with isoflurane before decapitation. The skin covering the skull was cut longitudinally with a surgical blade (Figure 3A) and the two skin flaps were held with the index and thumb fingers. After removing the surface connective tissue above the eyes (Figure 3B), optic nerves were separated from the eyeballs by carefully cutting with surgical scissors (Fine Iris Scissors, FST, 14106-09) into the eye socket behind the eyeballs (Figure 3C). Care was taken not to damage the eyeballs. The exit tips of the nerve heads were visible as white dots at the back of each eyeball after the separation (Figure 3D). Next, the skull was cut with surgical scissors and carefully removed to expose the brain (Figure 3E). Then the brain was pulled back to slowly extract the nerves out of the optic canals (Figure 3F). Importantly, the nerves should slip out easily without any stretching. Once pulled out, the nerves were flushed with artificial cerebrospinal fluid (ACSF) to straighten them on the ventral surface of the brain (Figure 3G). Using spring scissors (Cohan-Vannas Spring Scissors, FST, 15000-02) the chiasm was cut as depicted in Figure 3G; leaving the nerves still attached to half of the chiasm by which then the nerve pair was lifted (Figure 3H) and transferred to the recording chamber.

### Recording Setup Preparation

**Figure 4:**
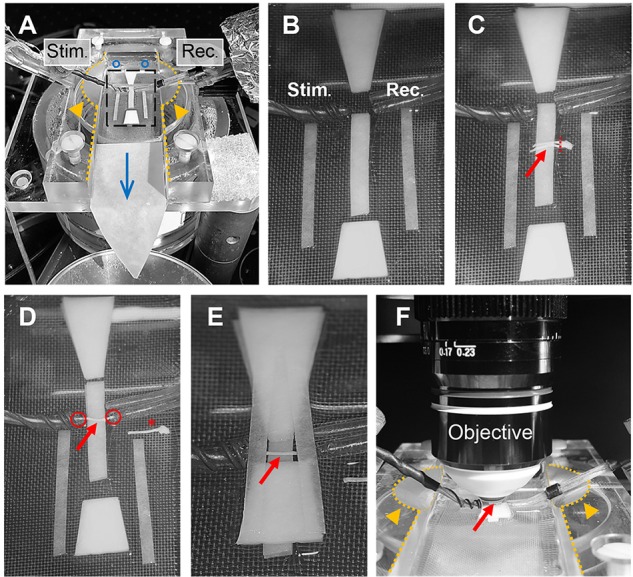
Optic nerve preparation in setup for electrophysiology and imaging. **(A)** Photograph of the recording chamber with stimulation (Stim.) and recording (Rec.) electrode. Orange arrow heads highlight the modification to the Haas top unit of the chamber; i.e., the inside edges (dotted lines) are milled down in a 45° angle to create better access for electrodes and objective, see also **(F)**. Blue circles and arrow indicate location of ACSF inflow into the chamber and direction of flow, respectively. Black rectangle outlines the region magnified in **(B)** showing the arrangement of filter papers around the electrodes. **(C)** The optic nerves (red arrow) are placed on the central filter paper strips (two layers) with the chiasm facing the recording electrode. One nerve is carefully cut from the chiasm (dashed red line) and moved towards the electrodes. **(D)** Depicted is how the nerve is positioned on the filter paper strips between the electrodes with each nerve end inserted into the suction electrodes (red circles). The other nerve is placed on the side as backup (red asterisk). **(E)** Organization of the remaining filter papers above and below the electrodes before **(F)** placing the objective above the nerve (red arrow) by dipping into the created ACSF pool. Orange arrows heads indicate the modification (milling) of the inside edges of the chamber (dotted lines).

Optic nerve recordings and imaging were performed in an interface recording chamber (Harvard apparatus, Base unit (BSC-BU, 65-0073) and Haas top (BSC-HT, 65-0075)) with custom-made modifications to the Haas top unit: the inside edges of the chamber were milled down in a ~45° angle to ~2 mm which created adequate space for positioning both suction electrodes and the objective (see Figures 4A,F). The recording chamber was continuously perfused with ACSF (containing in mM: NaCl, 126; KCl, 3; CaCl_2_, 2; NaH_2_PO_4_, 1.25; NaHCO_3_, 26; MgSO_4_, 2; D-glucose, 10; pH 7.4) which was constantly bubbled with 95% O_2_ and 5% CO_2_, and gravity-fed into the recording chamber at a flow rate of ~6 ml/min. Temperature was maintained at 37°C using a temperature controller (npi electronics, TC-10) with the sensor (TS-100, npi) attached inside the chamber. To reduce bubble formations inside the recording chamber, which could become an issue during imaging, the ACSF glass bottle was additionally preheated (during bubbling) in a plexiglass-walled (for better visibility) water bath (Julabo) at 37°C. The custom-made suction electrodes (Stys et al., 1991; Figures 4A–F) were back-filled with ACSF and positioned in the chamber using micro-manipulators (Mini 25, Luigs & Neumann).

The excised optic nerve pair was placed on filter paper (Macherey-Nagel, MN 615) strips inside the chamber as depicted in Figure 4C. Before starting the experiment, filter paper strips were prepared by cutting two strips of ~2 mm width, two strips of ~1.5 mm width and two trapezoid-shaped filter paper stacks (four layers) and arranged in the recording chamber as shown in Figure 4B. Filter papers were rinsed thoroughly with ACSF to remove any dust particles and the chamber was freed from bubbles before nerve preparation. After the excised nerve pair was placed on the central 2 mm filter paper strips (two layers) with the chiasm facing the recording electrode, one nerve was carefully cut from the chiasm using spring scissors (Figure 4C). The second nerve still attached to the chiasm was gently moved to the side and served only as a backup in case the first nerve was damaged during preparation (absence of CAP response) or lacked sensor expression due to a failed intravitreal injection. The nerve on the filter papers was positioned between the electrodes and each nerve end was inserted into the suction electrodes by slowly pulling back the plunger of the ACSF-filled syringes (attached to the electrodes via tubing; Figure 4D). The nerve ends should fit tightly into the electrodes and were not inserted more than 0.5 mm. The two trapezoid-shaped filter paper stacks (four layers) were carefully placed on the 2 mm filter paper strips above and below the nerve, leaving a ~1 mm gap above and below the electrodes (Figure 4E). Then the two 1.5 mm filter paper strips (single layers) were placed across the electrodes as shown in Figure 4E. This arrangement created a steady ACSF pool around the nerve and stabilized the optic nerve for subsequent imaging. Then the objective (working distance 2 mm) was positioned above the nerve by dipping it into the ACSF pool (Figure 4F). The imaging plane of the objective was focused on the nerve surface by visual control using a wide-field camera (installed in a separate optical path of the microscope). Finally, the syringes connected to the suction electrodes were carefully detached to release the pressure on the nerve ends which minimizes drift while imaging later. The nerve was allowed to equilibrate in this arrangement with the microscope objective in place and continuous ACSF flow for at least 30 min before starting the experiments.

### Optic Nerve Electrophysiology and Two-Photon Imaging

#### Nerve Stimulation and CAP Recording

**Figure 5:**
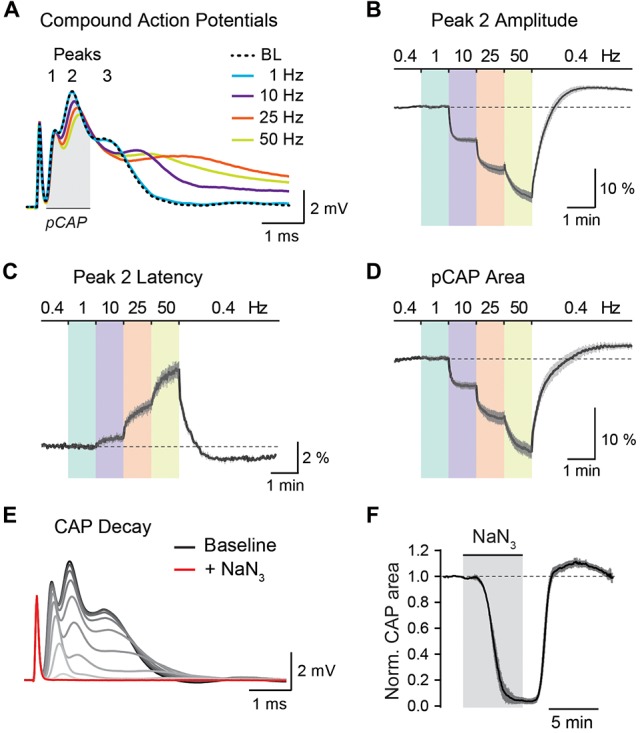
Analysis of optic nerve CAP recordings. **(A)** Example traces of the three-peak shaped CAP responses at 0.4 Hz baseline (BL) and at 1, 10, 25 and 50 Hz nerve stimulations. CAP traces of 1–50 Hz are taken from the end of a 1 min stimulus train. **(B,C)** Average time course of relative changes in peak 2 amplitude **(B)** and peak 2 latency **(C)** during the stepwise increase in stimulation frequency. Changes (in %) are presented relative to the 0.4 Hz BL recordings. Note that with higher stimulation frequencies, CAP peak amplitude decreases and peak latency increases. **(D)** Relative changes (in %) in partial CAP (pCAP) area with increasing stimulation frequency. The pCAP area is defined as the area under the curve (AUC) of the first two CAP peaks (indicated by the gray area in **(A)**) integrating changes in amplitude and latency. **(E)** Gradual decline of the CAP response (shown in 15 s intervals) to full conduction block (red trace) during inhibition of mitochondrial respiration with NaN_3_ (5 mM). **(F)** Average time course of the relative CAP area decay and recovery when challenged with NaN_3_ (5 mM) for 6 min. CAP area is normalized to BL before pharmacological treatment. In **(B–D,F)** data is represented as means ± SEM, *n* = 5 nerves from five animals.

Throughout all initial adjustments and during the equilibration phase (see above), the optic nerve was stimulated every 10 s and the evoked CAP response (Figure 5A) was monitored. Stimulations (square-wave current pulses of 50 μs and 0.8 mA) were triggered using a stimulus generator (STG 4002-1.6 mA, Multichannel Systems) which was connected to the stimulation electrode and controlled by the computer software MC_Stimulus. The recording electrode was connected to a miniature pre-amplifier (2× gain MPA, Multichannel Systems) and a data acquisition system (USB-ME16-FAI, Multichannel Systems). The signal was amplified 200 times, filtered at 30 kHz, and acquired at 50 kHz using the acquisition software MC_Rack (Multichannel Systems; RRID:SCR_014955). Stimulating the nerve with 0.8 mA elicited a maximal CAP response, meaning that all optic nerve axons are likely to have been excited (Stys et al., 1991). During the equilibration phase (especially after releasing the suction pressure from the nerve) the CAP amplitude typically dropped by 10%–20% while the nerve was adjusting to the electrodes. However, the CAP response normally became stable within 15–20 min.

#### Axonal Sensor Imaging

Optic nerve axons expressing the FRET-based ATP sensor (FRET pair: mseCFP and cp173-mVenus; Imamura et al., 2009) were imaged with a custom-built two-photon laser scanning microscope (Mayrhofer et al., 2015) using a Ti:Sapphire laser (Chameleon Ultra II; Coherent) at 870 nm wavelength and equipped with a 25× water immersion objective (XLPLN 25×/1.05 WMP2, Olympus). Fluorescence emission was detected with two GaAsP photomultiplier modules (Hamamatsu Photonics, H10770PA-40 SEL) using a dichroic beam-splitter (506 nm edge, BrightLine; Semrock, FF506-Di03-25 × 36) and two band-pass filters 545/55 nm (yellow channel) and 475/50 nm (blue channel; BrightLine; Semrock, FF01-545/55-25, FF02-475/50-25). Image acquisition was controlled by the MATLAB (RRID:SCR_001622) software ScanImage (RRID:SCR_014307; Pologruto et al., 2003). Laser power was adjusted between 5 and 15 mW for axonal sensor imaging. Initial overview stacks of axonal sensor expression were acquired at a resolution of 512 × 512 pixels. For sensor imaging during nerve stimulations, frames were acquired 15 to 20 μm below nerve surface with 8–10× digital zoom at 256 × 256 pixels and a pixel dwell time of 6.4 μs and images of both channels were collected simultaneously with two detectors every 2 s. CAP recordings and metabolite sensor imaging were synchronized using a TTL trigger driven by the stimulus generator to initiate both acquisitions (electrophysiology and imaging) as well as the stimulation protocol.

#### Stimulation Protocol and Pharmacology

We measured relative ATP level changes upon axonal stimulations using the following protocol: The optic nerve was first stimulated at 0.4 Hz for 1 min to acquire baseline (BL) values and was followed by a stepwise increase in stimulation frequency to 1, 10, 25 and 50 Hz, with each stimulus train lasting 1 min. A recovery period of 4 min with 0.4 Hz stimulations was added at the end of the stimulus sequence. During BL/recovery periods CAP responses were acquired every 2.5 s whereas during the high frequency trains CAPs were collected every second. Sensor images were acquired every 2 s throughout the entire 9 min stimulation protocol. This sampling rate is in line with the expected kinetics of the relative ATP level changes (Trevisiol et al., 2017) and avoids unnecessary bleaching. In a separate set of experiments, we pharmacologically blocked mitochondrial respiration using 5 mM NaN_3_ (Sigma-Aldrich, S2002). The drug was prepared in a separate ACSF bottle, bath applied to the nerve for 6 min and followed by a washout period for about 15 min. Before, during and after drug application sensor images and CAPs were acquired every 2 and 2.5 s, respectively.

The nerve could be studied for several hours with different brief stimulation protocols without any major changes in conduction performance. However, care should be taken if stimulation protocols (e.g., prolonged high frequency stimulations above 50 Hz) or pharmacological challenges (e.g., chemical ischemia) are applied that critically compromise axonal conduction and integrity. After such interventions the experiment should be concluded if reliable and reproducible measurements are desired.

#### Analysis of CAP Recordings and Sensor Imaging

The stimulus-evoked CAP response typically has three peaks, most likely representing subgroups of axons with different conduction speeds, and the overall area under the curve (AUC) of the CAP response is proportional to the total number of excited axons (Stys et al., 1991; Baltan et al., 2008; Saab et al., 2016). During high-frequency stimulations, CAP peak amplitudes generally decreased whereas peak latencies increased (Figure 5A). These changes were best assessed for the second and most prominent CAP peak (Figures 5B,C). Additionally, we measured relative changes in the partial CAP (pCAP) area (Figure 5D), i.e., the AUC of the first and second CAP peaks, typically in a range of 1 ms from the beginning of the CAP response as indicated in Figure 5A. The pCAP area integrates changes in amplitude and latency of the first two CAP peaks (Figure 5C), which likely derive from large and medium-sized axons that are reliably detected with conventional laser scanning microscopy (axonal diameters larger than ~0.5 μm). Relative changes in CAP amplitude, latency and AUC were calculated by normalizing values to BL prior to high-frequency simulations or NaN_3_ application. The normalized time course traces from nerve recordings were pooled, and data is presented as means ± SEM. CAP recordings were analyzed using a custom-written MATLAB script, which we provide for download from https://github.com/EIN-lab/CAP-analysis. However, the MATLAB script would need minor adjustments if other acquisition software formats than MC_Rack (multichannel systems; RRID:SCR_014955) are used.

An imaging time-series of the acquired mseCFP and mVenus channels was collected during each imaging experiment. The average signal intensity (of all axons per field of view) of each channel was extracted using ImageJ (RRID:SCR_003070; Schneider et al., 2012). For every acquisition timepoint the ratio of mVenus/mseCFP was calculated, and the time course of the ratios was normalized to the corresponding BL. The normalized time courses were pooled per animal and presented as means ± SEM. For comparisons of relative ATP level changes during high-frequency stimulations or mitochondrial block with NaN_3_, the slopes of the normalized time courses were calculated by linear fitting using GraphPad Prism (RRID:SCR_002798) and presented in summary plots as means ± SD. The ATP decay and recovery rates of the NaN_3_ experiments were determined from linear fits between 25% and 75% of the normalized decay and recovery curves. For all experiments only one nerve per animal was used and *n* represents number of animals.

## Results

**Figure 6:**
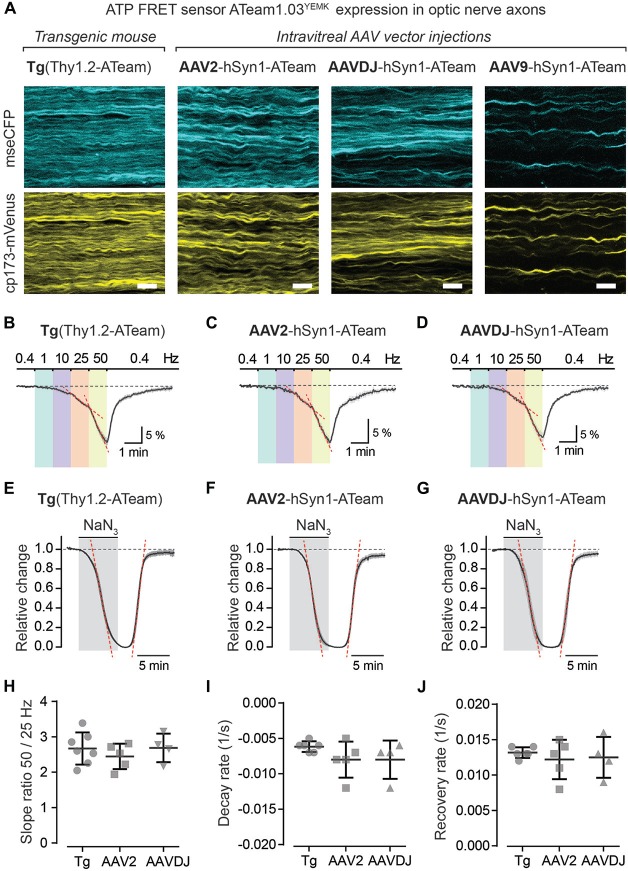
Axonal ATP sensor imaging following intravitreal AVV vector injections. **(A)** Example two-photon scan images of axons expressing the ATP sensor (ATeam1.03^YEMK^) in acute optic nerve preparations from a transgenic mouse [B6-Tg(Thy1.2-ATeam1.03^YEMK^)AJhi] and from mice with intravitreal injection of AAV vectors [hSyn1-ATeam1.03^YEMK^-WPRE-hGHp (A)]. Efficacy of sensor expression (number of axons) differs between the AAV vector serotypes tested, with AAV2 and AAVDJ revealing abundant expression whereas with AAV9 only very sparse axonal expression is detected. Both channels, mseCFP (blue) and cp173-mVenus (yellow), of the FRET sensor are depicted. Scale bars 10 μm. **(B–D)** Average time course of relative changes in axonal ATP levels upon stepwise increase in stimulation frequency in nerves from transgenic mice (*n* = 7; **B**) and from nerves following intravitreal AAV injections of serotypes AAV2 (*n* = 5; **C**) and AAVDJ (*n* = 4; **D**). Changes (in % ± SEM) are presented relative to the 0.4 Hz BL. **(E–G)** Relative ATP level changes in axons during and after mitochondrial respiration block, by 5 mM NaN_3_ bath application for 6 min, in nerve preparations from transgenic mice **(E)**, AAV2 **(F)** and AAVDJ **(G)** injected mice. To evaluate kinetics of ATP level changes (i.e., decay and recovery rates) the time course of the Venus/CFP ratios is normalized to BL (before NaN_3_ application; set as 1) and to the plateau level reached with NaN_3_ (set as 0). Depicted are averaged time courses from 4 to 7 nerves ± SEM. **(H)** Axonal ATP levels drop faster (i.e., increase in ATP consumption) with higher stimulation frequencies **(B–D)** and is exemplified here by calculating the ratios of the ATP level decline slopes (red dashed lines in **B–D**) upon 50 and 25 Hz stimulations. A two-fold increase in stimulation frequency reveals on average a 2.7 ± 0.4 (Tg), 2.4 ± 0.4 (AAV2) and 2.7 ± 0.4 (AAVDJ) fold increase in ATP level drop. No overt differences between groups, *n* = 4–7 ± SD, Welch’s *t-test*. **(I,J)** Summary plots of axonal ATP level decay **(I)** and recovery **(J)** rates from NaN_3_ experiments (in **E–G**). Rates (per s) were determined by linear regression between 25% and 75% of the normalized decay and recovery curves (red dashed lines in **E–G**; *R*^2^ of linear fits ≥0.98). Average decay rates **(I)** are −0.006 ± 0.0003 (Tg), −0.008 ± 0.001 (AAV2) and −0.008 ± 0.001 (AAVDJ) and average recovery rates **(J)** are 0.013 ± 0.0003 (Tg), 0.012 ± 0.001 (AAV2) and 0.012 ± 0.001 (AAVDJ). No overt differences between groups, *n* = 4–7 ± SD, Welch’s *t*-test.

Using intravitreal delivery and virus-mediated expression of genetically encoded sensors in optic nerve axons, we provide a novel method to measure axonal metabolite dynamics by combining optic nerve electrophysiology with two-photon imaging (Figures 5, 6). We showcase this approach by imaging axonal ATP dynamics following intravitreal AAV vector injections and expression of the ATP sensor ATeam1.03^YEMK^ (Imamura et al., 2009; Figure 6). Three different AAV vector serotypes (AAV2, AAVDJ and AAV9) were tested and axonal ATP sensor expression (driven by the hSyn1 promoter) was assessed. We observed that transduction efficacy varied strongly between the serotypes used, which was easily judged by the overall abundance of axons expressing the ATP sensor during acute optic nerve imaging (Figure 6A, right panels). Three to six weeks after intravitreal injections, AAV2 and AAVDJ revealed ample axonal expression whereas with AAV9 vectors only very sparse axonal expression was achieved. For comparison, optic nerves from transgenic mice, with a developmental, pan-neuronal expression of the same ATP sensor, show very abundant axonal expression levels (Trevisiol et al., 2017; Figure 6A, left panel).

To test axonal ATP sensor performance, optic nerves were metabolically challenged with increasing stimulation frequencies (Figures 6B–D) and by pharmacologically blocking mitochondrial ATP production (Figures 6E–G). Here, we only focused on nerves from AAV2 and AAVDJ injected mice as they revealed reliable and reproducible axonal expression compared to AAV9. As expected, a stepwise elevation in stimulation frequencies from a 0.4 Hz BL acquisition to 1, 10, 25 and 50 Hz revealed a faster axonal ATP level decline with increasing stimulation frequencies. A steeper axonal ATP level drop likely indicates higher ATP consumption rates due to increasing frequency-dependent energy demands. For example, a two-fold increase in stimulation frequency from 25 Hz to 50 Hz caused on average a 2.6-fold increase in axonal ATP level depletion (Figure 6H). Similarly, CAP performance also declines with increasing stimulation frequencies which is best described by the drop in CAP amplitude and pCAP area (Figures 5B,D). Moreover, when mitochondrial respiration and thereby ATP production was transiently blocked using 5 mM NaN_3_ (bath applied for 6 min) axonal ATP levels rapidly declined (Figures 6E–G) leading to a complete nerve conduction block (Figures 5E,F). This was reversed by washout of NaN_3_ which quickly replenished axonal ATP back to BL levels and completely restored axonal firing. We did not observe overt differences in ATP sensor dynamics between AAV-mediated sensor expressing nerves and transgenic nerves when comparing overall ATP decay and recovery kinetics (Figures 6I,J), which are also similar to previous results (Trevisiol et al., 2017). Hence, intravitreal injection of AAV vectors carrying genetically encoded sensors is a fast and reliable approach to drive functional sensor expression in optic nerve axons and by combining electrophysiology with two-photon imaging axonal metabolite dynamics can be studied.

## Discussion

Intravitreal delivery of AAV vectors to induce protein expression in RGCs is a widely used and safe technique causing no harm to retinal cells or alterations in retinal physiology (Martin et al., 2002; Hellström et al., 2009; Schön et al., 2015; Smith and Chauhan, 2018). We exploited this technique to drive metabolite sensor expression in optic nerve axons and to study activity-dependent axonal ATP dynamics in acute optic nerve preparations. This novel approach expands our previously described optic nerve imaging method (Trevisiol et al., 2017), allowing more flexibility to express and study dynamics of any biosensor of interest in optic nerve axons. Using our protocol the many established metabolite sensors, such as those for monitoring intracellular glucose (Takanaga et al., 2008), lactate (San Martin et al., 2013), pyruvate (San Martin et al., 2014a) or NADH (Hung et al., 2011; Zhao et al., 2011, 2015), when packed into appropriate AAV vectors, could be reliably expressed and studied in optic nerves from wildtype or genetically modified mice at different ages.

Long-term white matter integrity critically depends on oligodendroglial support functions (Griffiths et al., 1998; Lappe-Siefke et al., 2003; Snaidero et al., 2017) and recent studies have highlighted that myelinated axons may well receive metabolic support in the form of lactate from glycolytic oligodendrocytes (Fünfschilling et al., 2012; Lee et al., 2012; Saab et al., 2016). Using our versatile AAV-mediated approach to investigate metabolite sensors dynamics in myelinated axons may help to further resolve cellular and molecular mechanisms underlying oligodendroglial support functions in regulating axonal energy homeostasis. For example, axonal metabolite dynamics could be studied in mouse mutants with oligodendrocyte- or myelin-specific defects that develop axonal pathology (Griffiths et al., 1998; Lappe-Siefke et al., 2003; Kassmann et al., 2007; Lee et al., 2012; Saab et al., 2016) to better understand whether and how axonal metabolite homeostasis may be perturbed before the onset of axonal degeneration. Moreover, establishing biosensor expression in oligodendrocytes, ideally with a similar viral-mediated approach although technically more challenging, would be a significant expansion of our current optic nerve recording/sensor imaging method to further interrogate the axon-glial unit.

In optic nerves action potential propagation is well-maintained when exogenous lactate or pyruvate is supplied as the main energy source (Brown et al., 2001). And during periods of low glucose availability or aglycemia, lactate supply from astrocytic glycogenolysis becomes critical in sustaining optic nerve conduction (Wender et al., 2000; Brown et al., 2004; Tekkök et al., 2005). Inhibiting mitochondrial respiration with sodium azide in the presence of 10 mM glucose quickly diminishes axonal ATP levels and completely blocks nerve conduction (Figures 5, 6), suggesting that optic nerve axons are primarily fueled by oxidative phosphorylation (Trevisiol et al., 2017). On the other hand, axonal conduction in corpus callosum slice preparations is not well-preserved when exogenous lactate or pyruvate is supplied during glucose deprivation (Meyer et al., 2018). Interestingly, in these aglycemic conditions callosal axons are sufficiently energized to fire action potentials when glucose is presented through the gap junction-coupled oligodendroglial network, suggesting that glucose and/or endogenously generated lactate is transferred to axonal compartments (Meyer et al., 2018).

However, it remains uncertain how intracellular metabolites such as glucose, lactate or pyruvate are distributed between astrocytes, oligodendrocytes and the axonal compartment in different white matter tracts. Do axons take up and metabolize glial-derived lactate during physiological activity or only during metabolic stress such as aglycemia or post-ischemia? Do glial cells provide primarily lactate/pyruvate, or could unphosphorylated glucose also be shuttled to fuel axonal energy metabolism? How does glycolytic activity in glial cells or axonal compartments increase during electrical activity? What are the signaling mechanisms regulating activity-dependent axon-glia metabolic coupling? In future studies, metabolite sensor imaging in white matter tracts will help to resolve how different cellular compartments interact and exchange metabolites. Measuring activity-dependent axonal metabolite dynamics in acute optic nerve preparations provides a powerful tool to address some of these challenging questions. This opens an exciting avenue for future studies to interrogate molecular and cellular mechanisms of white matter energy metabolism and axon-glial metabolic coupling.

## Author Contributions

AS designed the protocol and trained ZL. AS and ZL performed experiments, analyses, prepared figures and wrote the manuscript. MB provided software and JH provided resources. BW provided technical support, infrastructure and funding. All authors contributed to the final manuscript.

## Conflict of Interest Statement

The authors declare that the research was conducted in the absence of any commercial or financial relationships that could be construed as a potential conflict of interest.

## Footnotes

^1^https://www.uzh.ch/vvf/ordersystem/
